# A Case of Femoral Hernia, Incarcerated Fourteen Days

**Published:** 1856-03

**Authors:** S. Nelson Burgess

**Affiliations:** Resident Surgeon St. Joseph’s Hospital


### *A Case of Femoral Hernia, incarcerated fourteen days.* Operated upon by Dr. McClellan.

[Reported by S. Nelson Burgess, Resident Surgeon St. Joseph's Hospital.]

Margaret Mertz, æt. 45, housekeeper, a German by birth, was brought into the Hospital, on the 2d of January, 1856, complaining of extreme debility, great irritability of stomach, and a small lump in the groin. On examination, I found a tumor of the size of a small walnut in the femoral region. She stated that it had made its appearance ten days before, and since that time, she had not had a passage from her bowels; there was no pain or even tenderness, either in the immediate neighborhood of the tumor, or in any part of the abdomen, which was much swollen and tympanitic.

The tumor was quite hard, firm and rounded, feeling more like an enlarged gland than any thing else. I attempted to reduce it, but failed, causing no pain; in fact, it could be manipulated with any amount of force, without the slightest complaint from her. She was extremely pale and anæmic, tongue furred, with cold, clammy perspiration. Enemas of turpentine and ol. ricini were repeatedly given, but brought away no feculent matter. I then administered minute doses of calomel, and ol. ricini, ℥j., which were vomited half an hour after taken. On the 4th, she was seized with stercoraceous vomiting, and continued to vomit for two days, at intervals, in large quantities. She now became rapidly weaker, and on the 6th, Dr. McClellan, the attending surgeon, was sent for, and after a careful examination, operated upon her. There was nothing worthy of note about the operation, except the close adhesions between the sac and the intestine, caused by numerous bands extending between the two, rendering them almost like one substance, which required careful dissection; the orifice of the crural ring was but slightly constricted, but the intestine was closely attached by bands of lymph. The intestine, which was congested and dark colored, was returned without difficulty. Ten minutes after the operation, she had a copious evacuation of dark fœtid matter, and expressed herself infinitely better. For several subsequent days, constant and large passages weakened her considerably, but she soon recovered when they were checked, and was discharged in three weeks from the time of the operation, perfectly well.

The peculiarities of the case, were the extreme length of time, 14 days, during which the intestine was incarcerated, so that no passage occurred from the bowels, and the entire absence of tenderness or pain, or any symptoms of inflammation in the hernial tumor, or abdomen; these conditions with the hard, rounded feeling of the tumor, were calculated in a great degree, to perplex the diagnosis. It may have been that a portion of the circumference of the intestine was confined within the ring, so as to paralyze the action of the bowels, without being sufficiently constricted to cause active inflammation.

The case presented an intermediate condition between the active acute symptoms of a real strangulated hernia, and the more passive one of the condition denominated incarcerated. The patient exhibited no symptoms that made us fear her death, but increasing debility, but, in all probability, she would not have lived 24 hours, without the relief afforded by an operation.

